# Palmitic acid alone or combined with stearic and oleic enhances ruminal fiber degradation and alters microbiome composition

**DOI:** 10.3389/fmicb.2025.1624738

**Published:** 2025-08-15

**Authors:** Fernanda Batistel, Osvaldo Gonzalez, Austin Sears, Sharif Uddin Khan, Jonas de Souza

**Affiliations:** ^1^Department of Animal Sciences, University of Florida, Gainesville, FL, United States; ^2^Department of Animal Sciences, Utah State University, Logan, UT, United States; ^3^Perdue Agribusiness, Salisbury, MD, United States

**Keywords:** bacteria, fatty acids, phospholipidic membrane, rumen, fiber

## Abstract

**Introduction:**

Improving ruminal fiber degradation is a key focus for enhancing animal performance and reducing the environmental impact of ruminant production systems. While dietary fat is typically recognized for impairing ruminal fiber degradation, recent research suggests that specific fatty acids, such as palmitic, stearic, and oleic, may have the potential to improve it. Since palmitic, stearic, and oleic are major components of the membranes of ruminal mixed bacteria, we hypothesize that supplying these fatty acids in proportions that mimic bacterial composition will promote microbial flow and, consequently, improve fiber degradation.

**Methods:**

Diets were randomly assigned to 8 single-flow continuous culture fermenters arranged in a replicated 4 × 4 Latin square with 6 days of adaptation and 4 days of sampling. Treatments were: (1) a basal diet without supplemental fatty acids (CON); (2) the basal diet plus 1.5% of palmitic acid (PA); (3) the basal diet plus 1.41% of stearic acid and 0.09% of oleic acid (SO); and (4) the basal diet plus 0.48% of palmitic acid, 0.95% of stearic acid, and 0.075% of oleic acid (PSO). Data were analyzed using a mixed model considering treatment as a fixed effect, and period and fermenter as random effects.

**Results and discussion:**

Both PA and PSO diets improved fiber degradation, increased the flow of short-chain fatty acids, and tended to increase microbial flow compared to the other treatments. Although the supply of dietary fatty acids did not change the total lipid content, they did alter the membrane fatty acid profile. For example, PA and PSO increased the concentration of specific fatty acids, such as *anteiso* C15:0, in the bacterial cell membranes, while SO and PSO reduced unsaturated fatty acids compared to PA and CON. Additionally, PA and PSO diets influenced the bacterial community, increasing populations of *Fibrobacter* and *Prevotella* while reducing *Ruminococcus* and *Butyrivibrio*. Our results indicate that including palmitic acid or a combination of palmitic, stearic, and oleic acids in proportions resembling those found in ruminal mixed bacteria improved ruminal fiber degradation, likely by partially modulating the rumen bacterial community composition.

## 1 Introduction

Ruminant animals, such as cattle, rely primarily on the fermentation of carbohydrates into short-chain fatty acids (SCFA) in the rumen as their primary energy source (Tedeschi et al., 2023). Unlike other carbohydrates such as starch and sugars, fibrous materials—measured as neutral detergent fiber (NDF)—are broken down exclusively by microbial enzymes, mainly in the rumen (Flint et al., 2012). The efficiency of fiber degradation not only affects animal productivity, including milk and meat output, but also has major implications for environmental sustainability. Poor NDF degradation limits feed intake and reduces animal performance, including milk and meat production (Oba and Allen, 1999). It also increases the environmental impact of livestock systems by elevating enteric methane emissions per unit of animal production and contributing to greater methane and ammonia losses from manure (Hristov et al., 2011). Consequently, there is substantial interest among nutritionists, microbiologists, and other researchers in improving ruminal fiber digestibility to enhance milk and meat production and mitigate the environmental footprint of ruminant production systems (Adesogan et al., 2019; Knapp et al., 2014).

Among the dietary factors that affect ruminal fiber degradation, the inclusion of fat is recognized as a contributor. For decades, dietary inclusion of fat-rich ingredients has been acknowledged to negatively affect fiber digestibility (Palmquist and Jenkins, 2017). However, recent studies exploring the effects of specific fatty acids on animal physiology and production indicate the potential benefits of specific fatty acids, such as palmitic, stearic, and oleic, in improving total-tract fiber digestibility. For example, supplements rich in palmitic (de Souza and Lock, 2018), palmitic and stearic (Piantoni et al., 2015a,b), or palmitic and oleic acid (de Souza et al., 2021) have been shown to increase total-tract fiber digestibility in dairy cows. In continuous culture fermenters, Wenner et al. (2025) demonstrated that supplying palmitic acid at 0.85% of dietary dry matter resulted in the greatest NDF degradation compared to 0, 1.7, and 2.5%. Nevertheless, why these three fatty acids promote fiber digestibility remains unclear. In a recent review, Firkins et al. (2025) emphasized the need to better understand how specific fatty acids interact with microbial consortia and influence carbon partitioning within the rumen, as these interactions may play a key role in enhancing fiber degradation.

Non-rumen bacteria are known to utilize at least three distinct mechanisms to incorporate exogenous fatty acids into their cell membranes (Yao and Rock, 2017), which help maintain membrane homeostasis and enhance survival under different environmental conditions (Zhang and Rock, 2008). In species like *Escherichia coli*, exogenous fatty acids rapidly inhibit fatty acid synthesis and promote their incorporation into the cell membranes (van den Berg et al., 2024). One key advantage of utilizing exogenous fatty acids, rather than relying solely on *de novo* synthesis, is the conservation of carbon, which can be redirected to other cellular functions and favor microbial growth (Zhang and Rock, 2008). Incorporating exogenous fatty acids into ruminal bacterial membranes remains largely unexplored. However, it recognized that palmitic, stearic, and oleic acids are major fatty acids found in the cell mass of mixed ruminal bacteria, comprising approximately 22, 43, and 2.1%, respectively (Or-Rashid et al., 2007). Therefore, we hypothesize that providing a dietary combination of fatty acids that mimic palmitic, stearic, and oleic proportions in mixed rumen bacteria will enhance their incorporation into bacterial cell membranes, promoting bacterial growth and increasing fiber degradation. Thus, this study aimed to examine how altering the proportions of supplemental palmitic, stearic, and oleic acids influences the fatty acid profile of bacterial membranes, microbial flow, composition of the rumen bacterial community, and fiber degradation.

## 2 Methods

### 2.1 Experimental design and diets

Diets were randomly assigned to 8 single-flow continuous culture fermenters (Teather and Sauer, 1988), an *in vitro* system that mimics the overall ruminal conditions. The treatments were arranged in a replicate 4 × 4 Latin square design with four 10-day experimental periods, consisting of 6 days for diet adaptation and 4 days for sample collection. The basal diet contained 50% orchardgrass hay and 50% concentrate and was supplied at 40 g/day [dry matter (DM) basis] in two equal daily offers (0800 and 1,600 h). The basal diet was chosen based on previous studies that evaluated different nutrition strategies to modify fiber digestibility and rumen fermentation in continuous culture fermenters (Wenner et al., 2020; Roman-Garcia et al., 2021a; Li et al., 2022). Treatments were: (1) a basal diet without supplemental fatty acids (total basal fatty acids: 2.57% diet DM; CON); (2) the basal diet plus 1.5% (DM basis) of supplemental palmitic acid (PA); (3) the basal diet plus 1.41% stearic acid and 0.09% oleic acid (SO) in DM basis of supplemental fatty acids; and (4) the basal diet plus 0.48% of palmitic acid + 0.95% of stearic acid + 0.075% of oleic acid (PSO) in DM basis of supplemental fatty acids. Treatment 2 was used as a positive control based on our previous study (Sears et al., 2024), in which the inclusion of 1.5% palmitic acid to the diet increased NDF degradation by 3 percentage units compared with a basal diet without supplemental fatty acids. Treatment 3 replicated the proportion of stearic and oleic acids found in mixed rumen bacteria (Or-Rashid et al., 2007), while treatment 4 replicated the proportion of palmitic, stearic, and oleic acids found in mixed rumen bacteria (Or-Rashid et al., 2007). All fatty acid treatments were supplied using pure fatty acids (99% pure; Catalog nos. P0500, S4751, and O1008; Sigma-Aldrich). Dietary ingredients and nutrient supply are presented in Table 1. The concentrate was ground to pass a 2 mm screen (Wiley mill; Thomson Scientific, Philadelphia, PA), and the orchardgrass hay was pelleted. Concentrate and hay were weighed into labeled plastic cups, and then the fatty acid treatments were added and mixed with the diet. Cups were sealed and stored at 4°C before administration.

**Table 1:** Dietary ingredients and chemical compositions of diets.

Ingredient, % DM	Treatment^1^			
	CON	PA	SO	PSO
Orchard grass hay	50.0	49.3	49.3	49.3
Ground corn	18.6	18.4	18.4	18.4
Canola meal	15.0	14.7	14.7	14.7
Beet pulp	4.40	4.40	4.40	4.40
Wheat middlings	10.5	10.2	10.2	10.2
Palmitic acid 99.9%	–	1.50	–	0.48
Stearic acid 99.9%	–		1.41	0.95
Oleic acid 99.9%	–	–	0.09	0.075
Mineral and vitamin mix^2^	1.50	1.50	1.50	1.50
Nutrient Supply^3^, g/day (DM basis)				
NDF	16.2	16.2	16.2	16.2
Crude protein	5.92	5.92	5.92	5.92
Starch	8.80	8.80	8.80	8.80
Total dietary fatty acids, mg/day	1.03	1.62	1.62	1.62
Palmitic acid	140	740	140	332
Stearic acid	16	16	580	392
Oleic acid	204	204	240	236
Palmitic + Stearic + Oleic	360	960	960	960

1 The control (CON) was a basal diet composed of 50% orchardgrass hay and 50% concentrate (dry matter basis) without supplemental fatty acids. The control (CON) was a basal diet composed of 50% orchardgrass hay and 50% concentrate (dry matter basis) without supplemental fatty acids. The control (CON) was a basal diet composed of 50% orchardgrass hay and 50% concentrate (dry matter basis) without supplemental fatty acids. PA treatment supplied 1.5% of palmitic acid; SO treatment supplied 1.41% of stearic acid + 0.09% of oleic acid; PSO treatment supplied 0.48% of palmitic acid +0.95% of stearic acid + 0.075% of oleic acid.

2 Vitamin and mineral mix contained 35% dry ground corn, 25.0% white salt, 22% calcium carbonate, 9.1% Biofos (The Mosaic Co., Plymouth, MN), 4% magnesium oxide, 2% soybean oil, and <1% of each of the following: manganese sulfate, zinc sulfate, ferrous sulfate, copper sulfate, iodine, cobalt carbonate, and selenium.

3 Calculated from average nutrient composition and feeding rate.

### 2.2 Continuous culture system operation

The inoculation of the continuous culture fermenters and operation procedures were similar to previous studies (Lascano et al., 2016; Roman-Garcia et al., 2021a,b; Sears et al., 2024). Briefly, at the beginning of each period, ruminal content was collected before morning feeding (0630 h) from two rumen-cannulated cows fed a lactating diet. The cows received a diet of 50% forage and 50% concentrate for at least 30 days before rumen collection. The rumen digesta was collected from the ventral, central, and dorsal areas of the rumen and then filtered through double-layered grade 60 cheesecloth into pre-warmed 39°C containers. The containers were kept at 39°C in a pre-heated water bath and immediately transported to the laboratory. Ruminal fluid was homogenized and mixed with artificial saliva (Weller and Pilgrim, 1974) containing 0.4 g/L of urea in a 1:1 proportion and maintained at 39°C. The ruminal fluid plus artificial saliva mixture was poured into each fermenter until the overflow spout cleared. During the experiment, fermenters were maintained at 39°C, carbon dioxide (20 mL/min) was continuously infused to maintain anaerobic conditions, and the fermenters’ content was uninterruptedly stirred by a central paddle set at a speed of 50 rpm. Artificial saliva was continuously bubbled with carbon dioxide to maintain the anaerobic condition and was constantly delivered at a 10%/hour fractional dilution rate using peristaltic pumps. The pH in the vessels was automatically measured every 10 min, and values ranged between 6.23 and 6.79. On day 5 of each period, fermenters were dosed with 50 mg of ammonium sulfate enriched with 10% ^15^N (Catalog no. 348473, Sigma-Aldrich) for microbial flow quantification. Additionally, the same ammonium sulfate was added to the artificial saliva at 25 mg/L from day 5 until the end of the experiment for a desired enrichment of 0.2% atom excess. Samples of the outflow effluent were collected before the ^15^N infusion to be used as background for microbial flow calculations.

### 2.3 Sample collection and analysis

Diet samples were collected in the last 4 days of each period, composited by period, and dried in a forced-air oven at 55°C for 72 h. Outflow effluent was collected on ice on days 7–10 of each period to prevent further fermentation. Four hundred mL of outflow effluent per fermenter was frozen at −20°C and freeze-dried (FreeZone 12, Labconco). Dried diet and outflow effluent samples were ground with a Wiley mill (1-mm screen; Arthur H. Thomas) before analyses. Diet and outflow effluent were analyzed for DM (method 934.01; AOAC International, 2000), ash (method 942.05; AOAC International, 2000), and NDF (Van Soest et al., 1991) with the use of heat-stable amylase (Catalog no. FAA, Ankom Technology) and sodium sulfite (Catalog no. S0505, Sigma-Aldrich). The NDF values were corrected for ash. Dietary nitrogen was determined by the Kjeldahl method (method 988.05; AOAC International, 2000). Dietary starch was determined according to Hall (2009), and the fatty acid content was determined using the one-step method of Sukhija and Palmquist (1988) with adaptations (Lock et al., 2013). NDF degradation was calculated as follows:


$$\begin{array}{l} \mathrm{NDF}\;\text{degradation},\%=(\begin{array}{l} \mathrm{NDF}\;\text{in the diet},g/\mathrm{day}-\mathrm{NDF}\;\\ \text{in the outflow effluent},g/\mathrm{day} \end{array})\\ /\mathrm{NDF}\;\text{in the diet},g/\mathrm{day}\times 100 \end{array}$$

Twenty mL of effluent was added to a bottle containing 1 mL of 6 N HCl and then frozen at −20°C. Samples were centrifuged (15,000 × *g*, 4°C, 15 min), and the supernatant was used to quantify SCFA using a gas chromatograph (Nexis GC-2030, Shimadzu Corporation) equipped with a capillary column (30 m × 0.53 mm i.d., 0.50 μm phase thickness, Restek). Crotonic acid (Catalog no. 113018, Sigma-Aldrich) diluted in toluene was used as an internal standard, and chromatograph conditions were as follows: helium 1.7 mL/min; oven temperature was 110°C held for 2.1 min, which was then increased by 25°C/min to 200°C; flame ionization temperature 220°C; split injection ratio 1/20; injection volume, 1 μL. Peaks were identified by the comparison of retention times with SCFA standards (catalog nos. A6283, I1754, 15,374, 240,370, 129,542, and CRM46975, Sigma-Aldrich; 149,300,025 and 108,110,010, Thermo Scientific). In the method used, isovalerate co-elutes with 2-methylbutyrate, and the two could not be distinguished in the present study.

The fatty acid profile of bacterial membranes was separated and analyzed as previously described (Sears et al., 2024). Briefly, bacterial cells from the effluent (500 mL) were isolated by centrifugation. Samples were kept at 4°C overnight to allow the detachment of bacteria from the feed particles and then centrifuged at 3500 × *g* for 5 min at 4°C to remove eukaryotes and feed particles. Subsequently, the supernatant was centrifuged at 20000 × *g* for 30 min at 4°C and resuspended once with NaCl solution (0.9%) containing Tween 80 (1 g/L; catalog no BP338-500, Fisher Scientific) and twice with distilled water. An aliquot of the bacterial cell was reserved for nitrogen (N) analysis, and the remaining cells were frozen, and 500 mg was used to extract the lipids (Folch et al., 1957). Lipids were extracted using methanol, chloroform, and a 2% NaCl solution. Lipid classes were separated by a solid-phase extraction method using a vacuum manifold kit (Catalog no. RE28298-VM, Restek) and aminopropyl SPE columns (Catalog no. 60108–432, Thermo Scientific) (Agren et al., 1992). After separation, samples were dried under N flow and weighed to obtain the phospholipidic fraction. The fatty acid profile of the phospholipidic fraction was determined using the two-step method (Sukhija and Palmquist, 1988) and adaptations proposed by Lock et al. (2013). The fatty acid methyl esters (FAME) were prepared by adding 5% methanolic sulfuric acid to the samples. The FAME was filtered through anhydrous sodium sulfate, solvents were removed under nitrogen flux at 37°C, the FAME were weighed, and a 1% solution with n-hexane prepared on a weight basis. The *cis*-10 C17:1 (catalog no. H8896, Sigma-Aldrich) diluted in toluene was used as an internal standard.

Ammoniacal N in the outflow effluent was determined by colorimetric analysis (Chaney and Marbach, 1962). Bacterial cells and outflow effluent were analyzed for total N and ^15^N isotopes. Dried effluent samples (50 mg) were weighed, wetted with distilled water, adjusted with 10 *N* NaOH to a pH > 10, and dried at 90°C for 16 h to remove ammoniacal N (Hristov et al., 2001). Both bacterial and effluent samples were analyzed for ^15^N enrichment according to procedures described by Noftsger et al. (2003). The background ^15^N levels were subtracted from the ^15^N enrichment after ^15^N infusion to determine the atom percentage excess (APE) of ^15^N. Ammoniacal N flow (g/day) was calculated as the product of ammoniacal N concentration and total effluent flow. Non-ammonia N (NAN) flow (g/day) was determined by subtracting ammoniacal N flow from total N.

Bacterial N flow was calculated using the formula:


BacterialNflow,g/day=NANflow,g/day×1515NAPEof outflow effluent/15NAPEof bacteria

Bacterial N per NDF degradation was calculated by dividing bacterial N flow by NDF degraded, while rumen undegradable protein (RUP) and rumen degradable protein (RDP) were calculated as follows:


RUP,%=non−ammoniaN−bacterialN/NAN×100

RDP,%=100−RUP

Total genomic DNA was extracted from outflow samples using the bead beating plus column method Yu and Morrison (2004), and DNA was quantified using a Qubit Fluorometer. PCR was performed using universal primers flanking the variable 4 (V4) region of the 16S rRNA gene (Kozich et al., 2013). Samples were quantified with a Qubit fluorometer, pooled on an equimolar basis, and sequenced with MiSeq v3 kit (2 × 300 cycles, Illumina) according to the manufacturer’s protocol. All sequences were demultiplexed on the Illumina MiSeq system. Further, sequence processing was performed using mothur v1.45.1 (Schloss et al., 2009) following the protocol described by Kozich et al. (2013). Briefly, paired-end sequences were combined into contigs, and poor-quality sequences were removed. Bacterial sequences were aligned and classified using the SILVA 16S rRNA database (Pruesse et al., 2007). All sequences were clustered into operational taxonomic units (OTU) at 97% similarity using uncorrected pairwise distances and the furthest-neighbor method. OTU tables were first rarefied to the lowest sequencing depth across samples and then normalized to relative abundance (% of total sequences) for downstream analyses.

### 2.4 Statistical analysis

Parts of the microbiota statistical analyses were carried out in R (vegan package). Total bacterial community structure (Bray-Curtis) and composition (Jaccard) were calculated from normalized OTU data and visualized by non-metric multidimensional scaling (NMDS) plots. The PERMANOVA was run to determine the differences in community structure and composition between treatments by using the *adonis* function in *vegan*, with the Benjamini–Hochberg correction for multiple comparisons.

Data for NDF degradation, ruminal N metabolism, fatty acids, alpha diversity, and relative abundance were analyzed using the MIXED procedure of SAS v.9.4 (SAS Institute, Inc. Cary, NC) according to the following model:


$$Y_{\mathrm{ijk}}=\mu +p_{i}+f_{j}+T_{k}+e_{\mathrm{ijk},}$$

where *Y_ijk_* = variable of interest, μ = overall mean, *p_i_* = random effect of period (*i* = 1 to 4), *f_j_* = random effect of fermenter (*j* = 1 to 8), *T_k_* = fixed effect of treatment (*k* = CON, PA, SO, and PSO), *e_ijk_* = residual error. The normality of the residuals was checked with normal probability and box plots and homogeneity of variances with plots of residuals *vs*. predicted values. A protected least significant difference was used for mean separation. Significance was declared at *p* ≤ 0.05 and tendency at *p* ≤ 0.10.

## 3 Results

### 3.1 *In vitro* NDF degradation and SCFA

**Figure 1:**
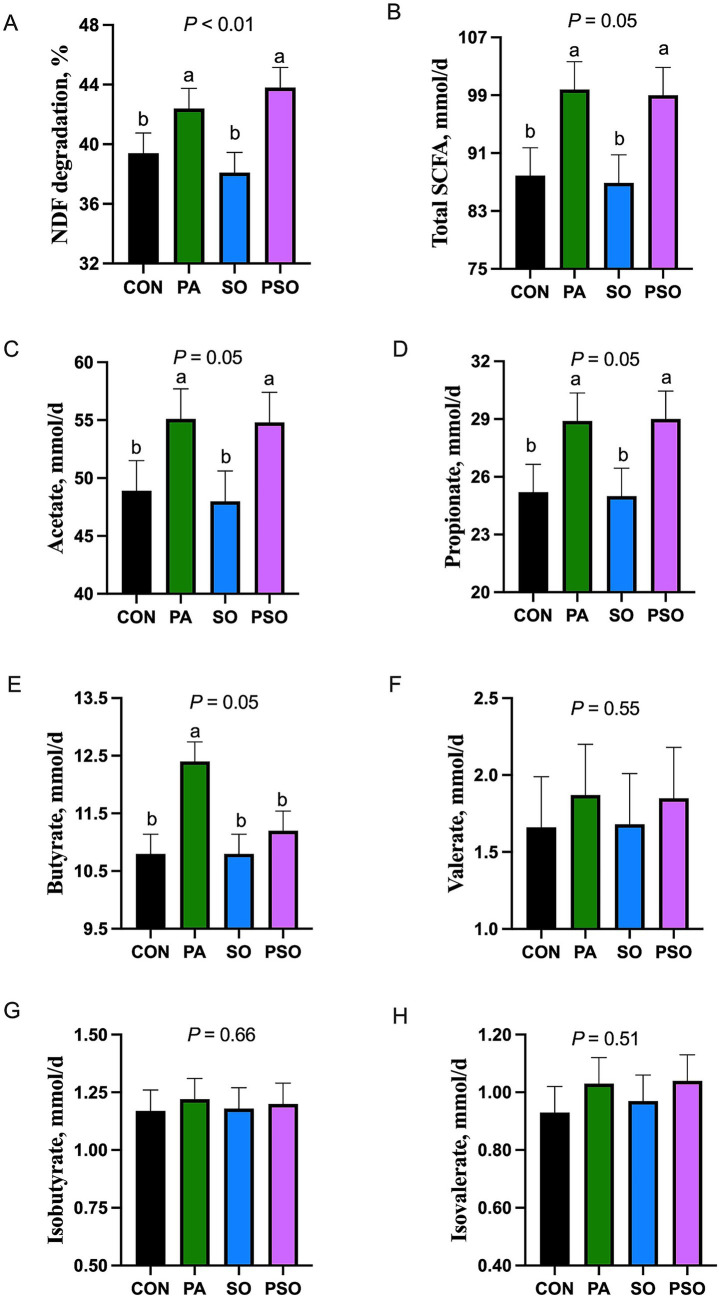
Effect of combinations of palmitic, stearic, and oleic acid on NDF degradation (A) and short-chain fatty acids (SCFA; B-H) flow in continuous culture fermenters. The control (CON) was a basal diet composed of 50% orchardgrass hay and 50% concentrate (dry matter basis) without supplemental fatty acids. PA treatment supplied 1.5% of palmitic acid; SO treatment supplied 1.41% of stearic acid + 0.09% of oleic acid; PSO treatment supplied 0.48% of palmitic acid + 0.95% of stearic acid + 0.075% of oleic acid. For treatment effect, means without a common letter differ (*p* < 0.05). Isovalerate co-elutes with 2-methylbutyrate, and the 2 could not be distinguished in the present study.

The degradation of NDF and the flow of total and individual SCFA are presented in Figure 1. Degradation of NDF was increased with PA and PSO compared with CON and SO (*p* < 0.01). Similarly, PA and PSO increased total SCFA flow (*p* = 0.05), acetate flow (*p* = 0.05), and propionate flow (*p* = 0.05) compared to CON and SO. PA increased butyrate flow compared with other treatments (*p* = 0.05). Treatments did not affect the flow of valerate, isobutyrate plus 2-methylbutyrate, and isovalerate.

### 3.2 Nitrogen flow

Bacterial N in the outflow effluent tended to be higher with PA and PSO compared to the other treatments (*p* = 0.08; Table 2). Additionally, PA and PSO increased rumen degradable protein and decreased rumen undegradable protein compared with CON and SO (*p* = 0.02). The treatments did not affect the flow of ammoniacal N, total N, bacterial N per unit of NDF digested, non-ammonia N, and non-ammonia non-bacterial N.

**Table 2:** Effect of palmitic, stearic, and oleic acid combinations on N metabolism in continuous culture fermenters.

Item	Treatments^1^				SEM	*p*-value^2^
	CON	PA	SO	PSO		
Ammoniacal N, mg/dL	17.7	18.7	18.1	17.8	2.39	0.69
Effluent N flow						
Total N, g/d	1.74	1.77	1.69	1.74	0.08	0.61
Bacterial N, g/d	0.68^b^	0.77^a^	0.68^b^	0.75^a^	0.05	0.08
Bacterial N per NDFD, g/kg^3^	72.2	69.1	71.0	68.9	6.51	0.90
Non-ammonia N, g/d	1.17	1.21	1.18	1.20	0.05	0.65
Non-ammonia non-bacterial N, g/d	0.49	0.44	0.50	0.45	0.06	0.32
Rumen degradable protein, %	57.6^b^	66.1^a^	56.8^b^	64.6^a^	2.73	0.02
Rumen undegradable protein, %	42.4^a^	33.9^b^	43.2^a^	35.4^b^	2.73	0.02

1 The control (CON) was a basal diet composed of 50% orchardgrass hay and 50% concentrate (dry matter basis) without supplemental fatty acids. The control (CON) was a basal diet composed of 50% orchardgrass hay and 50% concentrate (dry matter basis) without supplemental fatty acids. The control (CON) was a basal diet composed of 50% orchardgrass hay and 50% concentrate (dry matter basis) without supplemental fatty acids. PA treatment supplied 1.5% of palmitic acid; SO treatment supplied 1.41% of stearic acid + 0.09% of oleic acid; PSO treatment supplied 0.48% of palmitic acid +0.95% of stearic acid + 0.075% of oleic acid.

2 *p*-values refer to the ANOVA results for the main effect of fatty acid treatment.

3 NDFD, neutral detergent fiber degradation.

^a-c^For treatment effect, means without a common letter within the same row differ (*p* < 0.05).

### 3.3 Bacterial membrane fatty acid profile

The treatments did not alter total bacterial membrane flow (Table 3). The flow of *anteiso* C15:0 increased with PA and PSO compared to CON and SO (*p* = 0.02), while the flow of *anteiso* C13:0 tended to increase with PA and PSO compared to CON (*p* = 0.08). PA increased the flow of C17:0 (*p* = 0.03), while PA and PSO tended to increase the flow of C15:0 compared to CON (*p* = 0.07). C14:0 decreased with the fatty acid treatments compared to CON (*p* = 0.04), while PA decreased the flow of C18:0 compared to other treatments (*p* = 0.04). SO tended to decrease the flow of C13:0 compared to the other treatments (*p* = 0.06), while it tended to increase the flow of C18:1 *cis*-9 compared with PA and CON (*p* = 0.10). The flow of C18:1 *cis*-11 was decreased by PSO (*p* = 0.05) and the flow of C18:2 *cis*-9, *cis*-12 was decreased by SO (*p* = 0.05) compared to CON. We observed a tendency for SO and PSO to decrease the flow of C18:1 *cis*-7 (*p* = 0.08) and C18:3 *cis*-9, *cis*-12, *cis*-15 (*p* = 0.08) compared to PA and CON.

**Table 3:** Effect of palmitic, stearic, and oleic acid combinations on bacterial membrane fatty acid profile in continuous culture fermenters.

Item	Treatment^1^				SEM	*p*-value^2^
	CON	PA	SO	PSO		
Fatty acid, mg/d						
C9:0	0.97	0.71	0.83	0.67	0.14	0.18
C10:0	3.35	2.23	3.91	2.33	0.89	0.68
C11:0	3.86	3.39	3.07	2.28	0.68	0.14
C12:0	119	102	107	84.0	15.2	0.44
*iso* C13:0	24.8	20.3	19.3	15.7	4.79	0.18
*anteiso* C13:0	2.78^b^	3.73^a^	3.12^ab^	3.88^a^	0.44	0.08
C13:0	34.2^a^	29.9^a^	22.5^b^	29.1^a^	3.70	0.06
*iso* C14:0	52.3	47.3	39.8	39.4	7.93	0.13
*iso* C15:0	105	85.6	92.4	90.2	11.8	0.23
*anteiso* C15:0	302^b^	405^a^	300^b^	430^a^	38.3	0.02
C14:0	349^a^	287^b^	270^b^	262^b^	26.1	0.04
C15:0	270^b^	379^a^	306^ab^	343^a^	43.1	0.07
*iso* C16:0	41.9	37.1	31.9	30.2	4.90	0.24
C16:0	1,261	1,611	1,573	1,350	217	0.17
*iso* C17:0	23.0	18.9	18.2	17.0	2.43	0.38
C16:1 *cis*-9	50.1	49.4	43.1	42.7	4.59	0.23
C17:0	90.2^b^	108^a^	95.2^b^	91.4^b^	6.49	0.03
C18:0	204^a^	143^b^	222^a^	195^a^	20.2	0.04
C18:1*trans*-9	45.1	45.9	40.6	37.1	5.59	0.68
C18:1 *trans*-11	19.6	19.6	16.5	16.7	4.12	0.85
C18:1 *cis*-7	15.6^a^	13.2^ab^	10.3^b^	10.1^b^	2.31	0.08
C18:1 *cis*-9	298^b^	323^b^	412^a^	364^ab^	46.8	0.10
C18:1 *cis*-11	356^a^	309^ab^	281^ab^	216^b^	53.2	0.05
C18:2 *cis*-9, *cis*-12	248^a^	223^a^	125^b^	171^ab^	50.9	0.05
C18:3 *cis*-9, *cis*-12, *cis*-15	18.9^a^	17.3^a^	11.1^b^	12.7^b^	3.70	0.08
C19:0	6.25	5.59	4.92	504	0.66	0.49
C20:0	3.32	2.39	2.49	4.13	0.74	0.35
C22:0	2.89	1.91	2.27	3.15	0.64	0.54
C24:0	2.78	1.83	2.13	2.85	0.61	0.63

1 The control (CON) was a basal diet composed of 50% orchardgrass hay and 50% concentrate (dry matter basis) without supplemental fatty acids. The control (CON) was a basal diet composed of 50% orchardgrass hay and 50% concentrate (dry matter basis) without supplemental fatty acids. The control (CON) was a basal diet composed of 50% orchardgrass hay and 50% concentrate (dry matter basis) without supplemental fatty acids. PA treatment supplied 1.5% of palmitic acid; SO treatment supplied 1.41% of stearic acid + 0.09% of oleic acid; PSO treatment supplied 0.48% of palmitic acid +0.95% of stearic acid + 0.075% of oleic acid.

2 *p*-values refer to the ANOVA results for the main effect of fatty acid treatment.

^a-c^For treatment effect, means without a common letter within the same row differ (*p* < 0.05).

### 3.4 16S rRNA gene data acquisition and analysis

The sequencing of the bacterial 16S rRNA gene of the outflow effluent generated an average of 47,216 high-quality sequences per sample (Supplementary Table S1). Sequence coverage met a Good’s coverage greater than 99.5% for all samples, implying that sampling provided sufficient OTU coverage to describe the bacterial composition in each treatment accurately. There were no treatment effects for the number of sequences and Good’s coverage.

### 3.5 Richness, diversity, and composition of the bacterial communities

**Figure 2:**
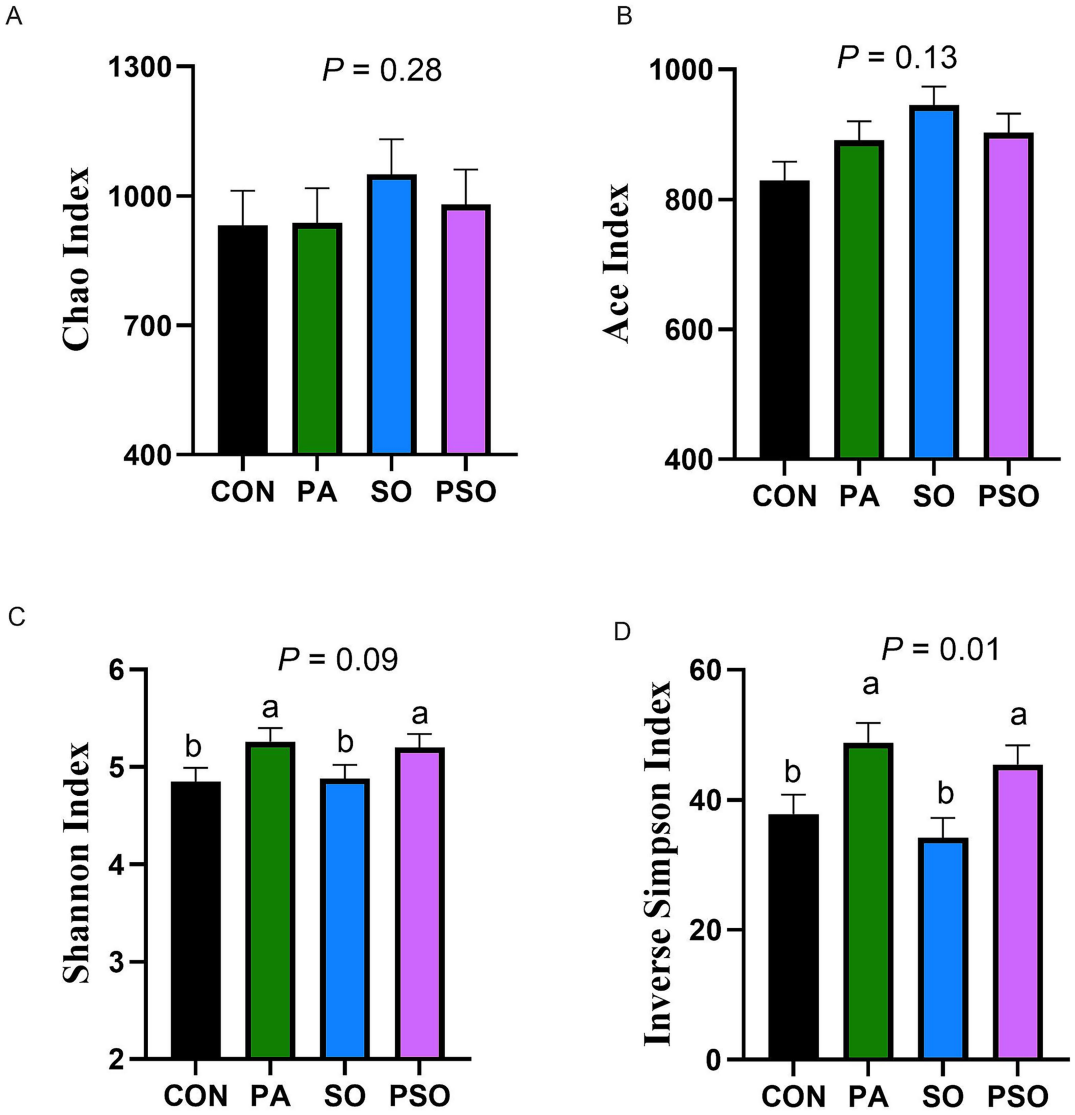
Effect of combinations of palmitic, stearic, and oleic acid on bacterial alpha diversity index for richness (Chao and Ace; A-B) and diversity (Shannon and Inverse Simpson; C-D) in continuous culture fermenters. The control (CON) was a basal diet composed of 50% orchardgrass hay and 50% concentrate (DM basis) without supplemental fatty acids. PA treatment supplied 1.5% of palmitic acid (% DM); SO treatment supplied 1.41% of stearic acid + 0.09% of oleic acid (% DM); PSO treatment supplied 0.48% of palmitic acid + 0.95% of stearic acid + 0.075% of oleic acid (% DM). For treatment effect, means without a common letter differ (*p* < 0.05). Error bars are the SEM.

The indices to assess richness (Chao and Ace) of the bacterial community were not affected by treatments (Figure 2). However, the diversity of the bacterial community increased with PA and PSO supplementation relative to the CON and SO treatments based on the Inverse Simpson index (*p* = 0.01). Similarly, we observed that PA and PSO tended to increase the Shannon index (*p* = 0.09) compared with CON and SO. For beta-diversity analysis, we did not observe a treatment effect on Bray-Curtis and Jaccard distances in the PERMANOVA analysis (Supplementary Table S2). The non-metric multidimensional scaling (NMDS) plot of the Bray–Curtis similarity index showed overlapping points (Supplementary Figure S1), indicating that treatments did not significantly affect the beta-diversity composition of the bacterial community.

**Figure 3:**
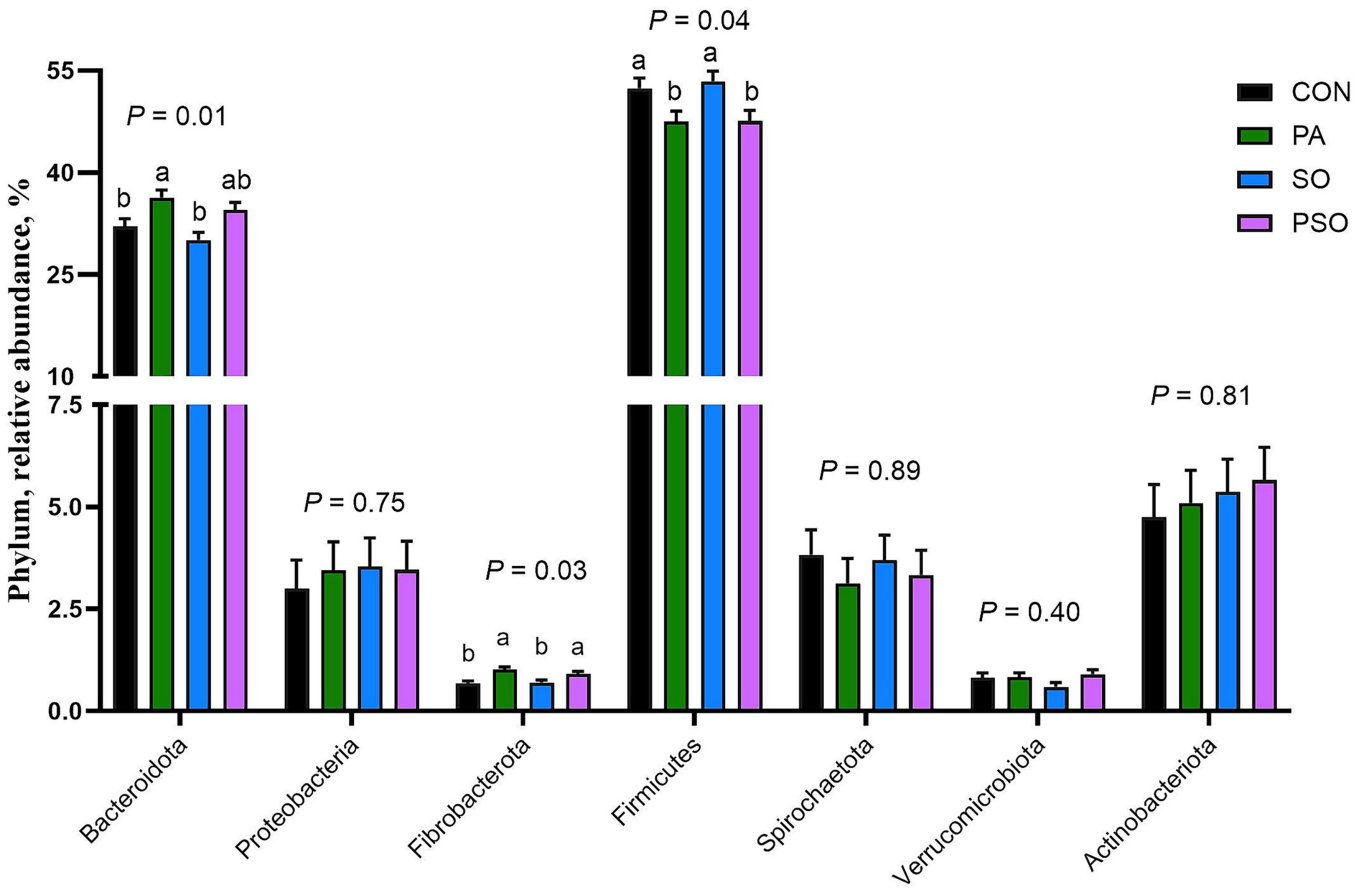
Effect of combinations of palmitic, stearic, and oleic acid on relative abundance of bacterial phylum in continuous culture fermenters. The control (CON) was a basal diet composed of 50% orchardgrass hay and 50% concentrate (dry matter basis) without supplemental fatty acids. PA treatment supplied 1.5% of palmitic acid; SO treatment supplied 1.41% of stearic acid + 0.09% of oleic acid; PSO treatment supplied 0.48% of palmitic acid + 0.95% of stearic acid + 0.075% of oleic acid. For treatment effect, means without a common letter differ (*p* < 0.05). Error bars are the SEM.

At the phylum level, a total of 14 bacterial phyla were identified; Figure 3 shows the seven most abundant, which together represent over 97% of the total relative abundance. Regardless of dietary treatment, the bacterial community composition was dominated by the phylum Bacillota (formerly Firmicutes; 50.2%) and Bacteroidota (formerly Bacteroidetes; 33.3%). PA increased Bacteroidota compared with the CON and SO (*p* = 0.01). PA and PSO increased Fibrobacterota (formerly Fibrobacteres) when compared with CON and SO (*p* = 0.05). In contrast, CON and SO increased Bacillota compared with PA and PSO (*p* = 0.04). The abundance of the phylum Actinobacteriota, Pseudomonadota (formerly Proteobacteria), Spirochaetota (formerly Spirochetes), and Verrucomicrobiota (formerly Verrucomicrobia) was not affected by treatments.

Twenty-three bacterial families represented over 90% of the abundance at the family level (Table 4). Prevotellaceae and Lachnospiraceae had the largest relative abundance across all treatments, accounting for 24.3 and 21.2% of total sequences, respectively. PA and PSO increased the relative abundance of Prevotellaceae compared with the other treatments (*p* = 0.01). In contrast, PA and PSO decreased the relative abundance of Lachnospiraceae compared with CON and SO (*p* = 0.05). PA and PSO increased the relative abundance of Fibrobacteraceae (*p* = 0.05) but decreased the abundance of Ruminococcaceae (*p* = 0.04) compared with CON and SO. The FA treatments did not affect the relative abundance of the other families identified from the 16S gene sequencing.

**Table 4:** Effect of palmitic, stearic, and oleic acid combinations on the relative abundance of ruminal bacterial families in effluent digesta.

Item	Treatments^1^				SEM	*p*-value^2^
	CON	PA	SO	PSO		
Prevotellaceae	22.5^b^	27.7^a^	21.6^b^	25.2^a^	1.38	0.01
Lachnospiraceae	22.2^a^	19.8^b^	22.8^a^	19.6^b^	1.25	0.05
Spirochaetaceae	3.83	3.12	3.69	3.31	0.45	0.88
Rikenellaceae	3.49	3.16	2.64	3.25	0.52	0.61
Ruminococcaceae	3.72^a^	2.89^b^	3.85^a^	3.15^ab^	0.33	0.04
Bifidobacteriaceae	2.84	2.90	3.81	4.19	1.54	0.34
Muribaculaceae	1.55	1.53	1.80	1.92	0.16	0.66
Christensenellaceae	1.23	1.50	1.06	1.49	0.15	0.41
Lactobacillaceae	3.01	2.30	3.13	2.23	0.42	0.29
Streptococcaceae	3.98	3.18	3.15	2.55	0.76	0.83
Veillonellaceae	3.89	3.65	4.91	4.49	0.43	0.54
F082	1.70	1.55	1.32	1.74	0.33	0.72
Atopobiaceae	2.39	2.58	2.99	2.83	0.44	0.46
Anaerovoracaceae	2.01	1.98	1.95	1.87	0.16	0.94
Oscillospiraceae	1.30	1.63	1.04	1.51	0.18	0.47
Selenomonadaceae	1.42	1.06	1.39	1.28	0.19	0.31
Acidaminococcaceae	1.31	1.24	1.12	1.15	0.15	0.60
Erysipelatoclostridiaceae	1.14	1.25	1.22	1.69	0.38	0.50
Fibrobacteraceae	0.68^b^	1.02^a^	0.69^b^	0.91^a^	0.08	0.05
Hungateiclostridiaceae	0.57	0.55	0.61	0.52	0.07	0.86
Erysipelotrichaceae	1.38	1.03	1.67	1.02	0.20	0.48
WCHB1-41_fa	0.77	0.79	0.56	0.84	0.16	0.41

1 The control (CON) was a basal diet composed of 50% orchardgrass hay and 50% concentrate (dry matter basis) without supplemental fatty acids. The control (CON) was a basal diet composed of 50% orchardgrass hay and 50% concentrate (dry matter basis) without supplemental fatty acids. The control (CON) was a basal diet composed of 50% orchardgrass hay and 50% concentrate (dry matter basis) without supplemental fatty acids. PA treatment supplied 1.5% of palmitic acid; SO treatment supplied 1.41% of stearic acid + 0.09% of oleic acid; PSO treatment supplied 0.48% of palmitic acid +0.95% of stearic acid + 0.075% of oleic acid.

2 *p*-values refer to the ANOVA results for the main effect of fatty acid treatment.

^a-c^For treatment effect, means without a common letter within the same row differ (*p* < 0.05).

We identified 91 bacteria genera with over 0.1% relative abundance. At the genus level, *Prevotella*, *Lachnospiraceae_unclassified* and *Butyrivibrio*, were the most abundant genera, representing 15.8, 7.35, and 4.45% of the total sequences, respectively (Table 5). PA increased the relative abundance of *Prevotella* (*p* < 0.01) *Prevotella*_Unclassified (*p* = 0.05; compared to the other treatments). PA and PSO increased the relative abundance of Prevotellaceae_UCG-003 (*p* = 0.02), Prevotellaceae_Ga6A1_group (*p* = 0.01), and *Fibrobacter* (*p* = 0.05) compared to CON and SO. We observed a tendency for PA and PSO to increase the relative abundance of Prevotellaceae_YAB2003_group (*p* = 0.08), Prevotellaceae_UCG-001_group (*p* = 0.07), and *Oribacterium* (*p* = 0.09) compared to the other treatments. Compared to CON and SO, PA and PSO decreased the relative abundance of *Ruminococcus* (*p* = 0.03). PA reduced the abundance of *Pseudobutyrivibrio* (*p* = 0.10) compared to the other treatments. PA and PSO reduced the abundance of *Butyrivibrio* (*p* = 0.01) and tended to decrease Lachnospiraceae_unclassified (*p* = 0.10) compared with CON. The relative abundance of the other bacterial genera was not affected by dietary treatments.

**Table 5:** Effect of palmitic, stearic, and oleic acid combinations on the relative abundance of ruminal bacterial genera in effluent digesta.

Item	Treatments^1^				SEM	*p*-value^2^
	CON	PA	SO	PSO		
Prevotella	14.4^b^	18.5^a^	13.6^b^	16.5^ab^	1.03	0.01
Butyrivibrio	4.81^a^	4.17^b^	4.53^ab^	4.32^b^	0.34	0.01
Prevotellaceae_Ga6A1_group	0.18^b^	0.36^a^	0.24^b^	0.42^a^	0.04	0.01
Prevotellaceae_UCG-003	2.42^b^	2.85^a^	2.33^b^	2.85^a^	0.24	0.02
Ruminococcus	3.11^a^	2.15^b^	3.24^a^	2.44^b^	0.24	0.03
Prevotellaceae_unclassified	2.68^b^	3.52^a^	2.75^b^	2.65^b^	0.24	0.05
Fibrobacter	0.67^b^	1.01^a^	0.67^b^	0.90^a^	0.08	0.05
Prevotellaceae_UCG-001	1.17^b^	1.39^a^	1.16^b^	1.40^a^	0.08	0.07
Oribacterium	0.82^b^	1.10^a^	0.92^b^	1.08^a^	0.14	0.09
Lachnospiraceae_unclassified	8.12^a^	6.79^b^	7.70^ab^	6.78^b^	0.53	0.10
Prevotellaceae_YAB2003_group	0.82^b^	1.17^a^	0.94^b^	1.14^a^	0.19	0.08
Pseudobutyrivibrio	0.97^a^	0.82^b^	0.99^a^	0.93^a^	0.06	0.10
Megasphaera	1.66	1.93	2.11	1.85	0.16	0.17
Acetitomaculum	2.54	2.73	3.70	2.23	0.52	0.19
UCG-004	0.42	0.29	0.39	0.30	0.04	0.22
Bifidobacterium	4.69	5.71	5.54	7.04	1.45	0.29
Prevotellaceae_UCG-004	0.41	0.27	0.36	0.33	0.05	0.29
Lactobacillus	2.96	2.27	3.08	2.21	0.67	0.30
Anaerovoracaceae_ge	0.88	0.77	0.93	0.71	0.15	0.37
WCHB1-41_ge	0.77	0.79	0.56	0.84	0.12	0.40
Christensenellaceae_R-7_group	1.20	1.46	1.03	1.45	0.69	0.41
Clostridia_UCG-014_ge	1.17	1.20	0.78	0.98	0.24	0.42
Olsenella	2.13	2.32	2.78	2.57	0.54	0.43
RF39_ge	0.68	0.87	0.51	0.86	0.19	0.45
UCG-010_ge	0.18	0.22	0.16	0.21	0.04	0.48
Lachnospira	0.25	0.22	0.27	0.21	0.03	0.53
Rikenellaceae_RC9_gut_group	3.39	3.05	2.51	3.14	0.69	0.57
NK4A214_group	0.64	0.74	0.49	0.65	0.14	0.63
Muribaculaceae_ge	1.53	1.51	1.78	1.89	0.46	0.68
Lachnospiraceae_NK3A20_group	1.17	1.03	1.10	1.14	0.09	0.68
F082_ge	1.70	1.55	1.32	1.74	0.60	0.72
Saccharofermentans	0.54	0.51	0.59	0.49	0.10	0.75
Lachnospiraceae_XPB1014_group	0.15	0.16	0.13	0.16	0.03	0.84
Treponema	3.71	3.04	3.57	3.20	0.85	0.88
Ruminococcaceae_ge	0.04	0.04	0.03	0.04	0.005	0.88
Firmicutes_unclassified	0.11	0.12	0.12	0.13	0.02	0.93
Mogibacterium	0.46	0.49	0.43	0.47	0.07	0.94
Ruminococcaceae_unclassified	0.41	0.46	0.43	0.42	0.10	0.95
Lachnospiraceae_ge	0.84	1.01	0.92	0.89	0.15	0.96

1 The control (CON) was a basal diet composed of 50% orchardgrass hay and 50% concentrate (dry matter basis) without supplemental fatty acids. The control (CON) was a basal diet composed of 50% orchardgrass hay and 50% concentrate (dry matter basis) without supplemental fatty acids. The control (CON) was a basal diet composed of 50% orchardgrass hay and 50% concentrate (dry matter basis) without supplemental fatty acids. PA treatment supplied 1.5% of palmitic acid; SO treatment supplied 1.41% of stearic acid + 0.09% of oleic acid; PSO treatment supplied 0.48% of palmitic acid +0.95% of stearic acid + 0.075% of oleic acid.

2 *p*-values refer to the ANOVA results for the main effect of fatty acid treatment.

^a-c^For treatment effect, means without a common letter within the same row differ (*p* < 0.05).

## 4 Discussion

Recent research indicates that certain fatty acids, including palmitic, stearic, and oleic acids, could enhance total-tract fiber digestibility (de Souza and Lock, 2018; Piantoni et al., 2015a,b; de Souza et al., 2021). Nevertheless, why these fatty acids improve fiber digestibility is not well understood. Therefore, this study aimed to examine how altering the proportions of supplemental palmitic, stearic, and oleic acids—major fatty acids found in rumen bacterial cells—influences the fatty acid profile of bacterial membranes, microbial flow, composition of the rumen bacterial community, and fiber degradation. To note, fatty acids are naturally present in commonly used dietary ingredients, including those used in this experiment. Ingredients such as forages, corn, and soybean meal contribute to the background supply of fatty acids, primarily in the form of unsaturated fatty acids (NASEM, 2021). For example, the basal diet in this study contained 0.35, 0.04, and 0.51% of diet DM as palmitic, stearic, and oleic acids, respectively. These background levels contribute to the total fatty acid supply and should be considered, along with the fatty acids added through treatment supplementation, when interpreting the results.

Our results show that PA and PSO increased fiber degradation by 4 percentage units compared with CON and SO, while SO did not affect fiber degradation compared with CON. Previous studies have suggested that the impact of fatty acids on total-tract fiber digestibility depends on the source of the fatty acid source (Weld and Armentano, 2017). dos Santos Neto et al. (2021) indicated that feeding cows with a palmitic acid-enriched supplement (1.81% diet DM; 85% palmitic, 2% stearic, and 7.5% oleic acid) increased total-tract fiber digestibility by 4.5 percentage units while feeding a prill containing mixed fatty acids (2.26% diet DM; 38% palmitic, 45% stearic, and 8% oleic acid) did not affect NDF digestibility compared to a control diet without supplemented fatty acids. Our previous research shows that when fatty acids were fed at 1.5% of the diet (DM basis), palmitic acid increased NDF degradation, stearic acid had no effect, and oleic acid reduced NDF degradation compared to CON in continuous culture fermenters (Sears et al., 2024). Compared with palmitic acid, fewer studies investigated the inclusion of stearic and oleic acid-enriched supplements into the diet of dairy cows. Interestingly, in our current study, when stearic and oleic acid were supplied together without palmitic acid, fiber degradation was not affected. Previously, feeding stearic acid-enriched supplements to dairy cows tended to increase total-tract fiber digestibility (Piantoni et al., 2015a,b) or not influence fiber digestibility compared with a diet without supplemental fatty acids (Boerman et al., 2017). Regarding oleic acid, increasing its dietary concentration from 0.68 to 0.98% of diet DM had no effect on NDF digestibility in dairy cows (de Souza et al., 2019). In contrast, our previous study using a continuous culture system showed that supplementing with an additional 1.5% oleic acid, raising the total dietary concentration from 0.70 to 2.2% of diet DM, reduced NDF degradation compared to the control diet (Sears et al., 2024). These findings suggest that the impact of oleic acid on rumen fermentation may be dose-dependent. Overall, the differences in fiber degradation observed in the current study may be attributed to shifts in microbial flow and community composition in response to the specific fatty acid profiles of the supplements.

Rumen fermentation and the resulting quantity and profile of SCFA produced depend on several factors, including the form and availability of substrates, the rumen environment, the composition of the microbial population, and the need to recycle reducing equivalents generated during fermentation (Russell, 2002; Ungerfeld, 2020). Interconversion of SCFA and their use in anabolic processes allow anaerobic bacteria to efficiently utilize metabolic intermediates (Firkins et al., 2006). When supplemental fat is fed, effects on SCFA concentrations have been inconsistent (Palmquist and Jenkins, 2017), likely due to differences in the fatty acid profile of the supplement. For example, de Souza et al. (2023) reported that feeding calcium salts of palm oil (1.8% diet DM; 48% palmitic, 38% oleic, 6% linoleic acid) increased ruminal concentrations of acetate and total SCFA compared to calcium salts of soybean oil (1.8% diet DM; 17% palmitic, 21% oleic, 55% linoleic acid), highlighting the importance of fatty acid composition in shaping fermentation outcomes. In our current study, PA and PSO increased the flow of acetate, propionate, and total SCFA, while PA specifically increased the flow of butyrate compared to the other treatments. In contrast, the supply of SO did not affect rumen fermentation parameters compared to the control. The increase in acetate flow observed with PA and PSO aligns with the improvement in fiber digestibility, which is known to be positively associated with acetate production (Chen et al., 2021). Similarly, Sears et al. (2024) reported that supplementation with palmitic acid, compared to stearic or oleic acid (all at 1.5% diet DM), increased propionate and total SCFA flow in continuous culture fermenters. Because the basal diet was identical across treatments (apart from the fatty acid profile), we can rule out differences in nutrient supply, particularly carbohydrate availability, as the cause of variation in SCFA flow. According to fermentation stoichiometry, carbon inputs must be reconciled with carbon outputs in the form of SCFA, microbial biomass, transport processes, and motility (Ungerfeld, 2020). *De novo* fatty acid synthesis is a significant carbon sink, consuming both carbon and reducing equivalents derived from fermentation (Hackmann and Firkins, 2015b). If bacteria instead incorporate exogenous fatty acids, they may redirect reducing equivalents into other sinks, such as cellular biosynthesis or SCFA elongation. One potential mechanism for disposing of excess reducing equivalents is the elongation of SCFA. For instance, inhibition of methanogenesis has been shown to increase the incorporation of hydrogen into longer SCFA rather than acetate (Ungerfeld, 2015). Acetate can also be used in the synthesis of butyrate via acetyl-CoA, and certain rumen bacteria can generate butyrate through nonclassical pathways that help balance redox status (Diez-Gonzalez et al., 1999; Hackmann and Firkins, 2015a). Overall, our results demonstrate that PA and PSO supplementation enhanced NDF degradation and SCFA flow, particularly acetate, propionate, and butyrate, whereas SO did not. This suggests that palmitic and oleic acids, rather than stearic acid, more effectively modulate rumen bacterial metabolism at the levels tested. Since all fatty acid supplements were provided in the same physical form and dose, the observed effects can be attributed to differences in their fatty acid composition. Future studies using carbon-labeled fatty acids are needed to confirm their specific roles in bacterial metabolism and membrane incorporation.

Our results indicate that PA and PSO tended to promote microbial flow compared to the other treatments. In mixed rumen bacteria, microbial growth typically achieves only one-third to two-thirds of the theoretical maximum because a substantial portion of available energy is diverted to non-growth and maintenance functions (Hackmann and Firkins, 2015b). While the effects of dietary carbohydrates and nitrogen on bacterial growth are well established, the role of individual dietary fatty acids remains less understood. Supplemental fat may enhance microbial protein synthesis by reducing protozoal predation on bacteria or by alleviating the energetic cost of *de novo* fatty acid synthesis (Hanigan et al., 2013). In our previous study, we found that supplementing palmitic acid (1.5% of diet DM) tended to increase bacterial nitrogen flow, whereas stearic and oleic acids at the same inclusion level did not (Sears et al., 2024). This suggests that the microbial response depends on both the fatty acid profile and feeding level. Exogenous fatty acids serve as precursors for membrane phospholipids, and their incorporation can support bacterial growth (Yao and Rock, 2017). In non-rumen bacteria like *Escherichia coli* and *Listeria monocytogenes*, supplying 18-carbon fatty acids has been shown to enhance membrane incorporation and growth rates, unlike shorter (<18C) or longer (>18C) fatty acids (Herndon et al., 2020; Flegler et al., 2022). Yao and Rock (2015) noted that many pathogenic bacteria rely on exogenous long-chain fatty acids from the host to reduce reliance on costly *de novo* synthesis, supporting growth in nutrient-limited environments. Because of the continuous outflow of solids and liquids in the rumen, microbes must reproduce rapidly to avoid washout (Van Soest, 1994). As proposed by Firkins et al. (2025), providing dietary fatty acids may offer bacteria membrane substrates, allowing them to conserve carbon and reducing equivalents for cell division. Our findings suggest that the supply of specific dietary fatty acids may enhance the microbial flow of mixed rumen bacteria by altering substrate availability and energy use for membrane functions.

The supply of exogenous fatty acids did not affect the total lipid concentration (average of 12.5% of DM). Previous reports have shown that total lipid content in the rumen bacterial mass ranges from 10 to 15% (Jenkins, 1993; Mitchell et al., 2023), and our observations for all the treatments fall within this range. Bacterial lipids are located in membranes and consist primarily of phospholipids, which contain a hydrophilic phosphate head group and a hydrophobic tail of two fatty acids (Gullett and Rock, 2021). Bacterial survival depends on membrane lipid homeostasis and the ability to adjust lipid composition in response to environmental conditions (Yao and Rock, 2017). The lack of treatment effects on total bacteria phospholipids is not surprising. The consistency in the phospholipid fraction across treatments was expected, as significant modifications to membrane fatty acids usually occur in response to environmental stressors, such as acidic conditions or temperature (Zhang and Rock, 2017), which were kept constant across treatments in our study. Additionally, bacterial cells tightly regulate the balance between lipid and macromolecular synthesis, ensuring that the membrane protein-to-lipid ratio remains constant across different growth rates (Parsons and Rock, 2013). Thus, our results align with previous literature, suggesting that changes in the bacterial fatty acid profile are more likely related to bacterial metabolism rather than to variations in the total lipid fraction.

The supply of exogenous fatty acids primarily influenced the composition of bacterial membrane lipids by increasing the abundance of *de novo* synthesized odd- and branched-chain long-chain fatty acids. Fatty acids in bacterial phospholipids can originate either from endogenous biosynthesis via the type II fatty acid synthesis (FASII) pathway or through the direct incorporation of exogenous fatty acids (Erwin, 1973). FASII is a modular, enzyme-based system in which acyl chains are elongated by two-carbon units, with intermediates bound to an acyl carrier protein (ACP). The final products, acyl-ACP molecules, are utilized in the synthesis of phosphatidic acid, the precursor for all bacterial phospholipids (Yao and Rock, 2017). This pathway is energetically costly; for instance, the synthesis of palmitate requires 8 acetyl-CoA, 14 NADPH, and 7 ATP molecules (Rock and Jackowski, 2002). To conserve carbon, many non-rumen bacteria have evolved mechanisms to utilize exogenous fatty acids, which are activated through one of three characterized systems: acyl-CoA synthetase, acyl-ACP synthetase, or fatty acid kinase (Yao and Rock, 2017). The metabolic fate of exogenous fatty acids and their interaction with FASII is highly species-dependent. In members of the order Lactobacillales (e.g., *Lactococcus*, *Streptococcus*), exogenous fatty acids can fully repress FASII, enabling complete reliance on environmental lipids for phospholipid biosynthesis. This repression occurs via both transcriptional regulation and biochemical feedback mechanisms that reduce malonyl-CoA synthesis, potentially through inhibition of acetyl-CoA carboxylase by acyl-ACP or acyl-phosphate intermediates (Lu and Rock, 2006; Jerga and Rock, 2009; Davis and Cronan, 2001). In contrast, Gram-positive bacteria such as *Staphylococcus aureus* and *Listeria monocytogenes* cannot bypass FASII inhibition entirely, due to their requirement for branched-chain fatty acids such as anteiso-15:0, which are not readily available in the host environment (Parsons et al., 2011; Zhu et al., 2010). In these taxa, exogenous fatty acids only partially suppress FASII, and inhibition of the pathway leads to the accumulation of short-chain acyl-ACP intermediates, depletion of free ACP, impaired phospholipid synthesis, and halted bacterial growth (Yao and Rock, 2017). This differential regulation underscores the complex interplay between external lipid availability and endogenous fatty acid metabolism and suggests that, in certain bacterial taxa exogenous lipid incorporation may shift carbon allocation, influence membrane fluidity and structure, and ultimately affect microbial competitiveness and community composition.

Rumen bacteria also synthesized *de novo* odd and branched-chain fatty acids using amino acids and SCFAs and incorporated them into their cell membrane (Fievez et al., 2012). In our study, although we did not observe treatment effects on >16-carbon even-chain fatty acids, our results indicate that PA and PSO increased the synthesis of odd-chain fatty acids (C13:0, C15:0, and C17:0) compared to other treatments. Linear odd-chain fatty acids are formed when propionyl-CoA, rather than acetyl-CoA, is used as the primer (Vlaeminck et al., 2006). Since we observed increased propionate flow with PA and PSO, the increase in linear odd-chain fatty acids may be linked to the greater propionate concentration. We also observed an increase in *anteiso* C13:0 and C15:0 with PA and PSO supplementation. The synthesis of *anteiso* fatty acids is driven by 2-methylbutyburate (Vlaeminck et al., 2006). Supply of isoacids (isovalerate, isobutyrate, and 2-methylbutyrate) in continuous culture fermenters resulted in higher ^13^C recovery in *anteiso* branch-chain fatty acids than *iso* odd-chain or *iso* even-chain branch-chain fatty acids highlighting the importance of 2-methylbutyrate for ruminal bacterial lipid synthesis (Mitchell et al., 2023; Roman-Garcia et al., 2021a,b). However, due to the co-elution of isovalerate with 2-methylbutyrate in our GC procedure, we could not assess whether the greater flow of *anteiso* fatty acids was associated with 2-methylbutyrate. Additionally, the composition of odd- and branched-chain fatty acids varies by bacterial taxa. Cellulolytic bacteria contain high levels of *iso* fatty acids, while higher proportions of *anteiso* and linear odd-chain fatty acids are associated with bacteria specialized in the fermentation of pectin and sugars (Vlaeminck et al., 2006). Therefore, changes in bacterial fatty acid flow may reflect alterations in the bacterial community in addition to substrate availability. Altogether, our data suggest that the supply of dietary fatty acids, particularly those containing palmitic acid or a profile that mimics the proportions of 16- and 18-carbon fatty acids in mixed rumen bacteria, may promote greater incorporation of exogenous fatty acids into the bacterial cell membranes, helping maintain membrane homeostasis and potentially conserving energy for other functions.

In our study, supplementation with PA and PSO increased the relative abundance of bacteria from the genera *Fibrobacter* and *Prevotella*, while reducing the abundance of *Ruminococcus* and *Butyrivibrio*. The genera *Fibrobacter* and *Ruminococcus* are well known for their cellulolytic activity, playing key roles in the degradation of cellulose in the rumen (Forano et al., 2008; Flint et al., 2008). Conversely, *Prevotella* and *Butyrivibrio* primarily contribute to the breakdown of non-cellulosic plant polysaccharides such as hemicellulose and pectin (Avguštin et al., 1997; Krause et al., 2003). The association between the increased abundance of *Fibrobacter* and *Prevotella* and the enhancement in fiber digestion may be explained by their ability to act synergistically within a microbial consortium. While *Fibrobacter* specializes in degrading crystalline cellulose through tightly associated surface-bound enzymes, it also releases soluble sugars that can be cross-fed to other members of the microbial community, including *Prevotella*. In turn, *Prevotella* can degrade other structural carbohydrates such as arabinoxylans and pectins, contributing to a more comprehensive breakdown of plant fiber. Additionally, the removal of fermentation intermediates and the production of growth-promoting metabolites by *Prevotella* may facilitate the activity and persistence of *Fibrobacter* in the rumen ecosystem. Another potential explanation for the observed microbial shifts is that the affected genera respond differently to the presence of exogenous fatty acids due to variations in their metabolic pathways. Previous studies have highlighted differences in fatty acid metabolism strategies among various non-rumen bacterial species (Yao and Rock, 2017), suggesting that the specific pathway used can influence how fatty acids are utilized or tolerated. Further investigation into the fatty acid metabolism pathways employed by the genera affected in the present study will help to clarify this hypothesis.

Supplementation with SO had a neutral effect on rumen fermentation or bacterial composition. We did not observe differences between the SO and CON treatments for fiber degradation or SCFA. Similarly, previous studies that supplemented dairy cows with stearic acid-enriched supplements did not observe changes in total tract fiber digestibility in dairy cows (Piantoni et al., 2015a,b; Boerman et al., 2017). Stearic acid is typically the predominant fatty acid reaching the duodenum, as it is the final product of rumen biohydrogenation of unsaturated fatty acids present in the diet (Jenkins et al., 2008). Additionally, in our current study, the supply of SO did not alter the bacterial composition or microbial flow compared to CON. A previous study reported that stearic acid had a neutral effect on rumen fermentation and bacterial composition, while oleic acid modified bacterial composition and reduced fiber degradation when purified supplements were fed at 1.5% of diet DM in continuous culture (Sears et al., 2024). This suggests that the feeding level may influence oleic acid’s impact on rumen function. Our data indicate that 18-carbon fatty acids may only positively affect rumen fermentation when combined with palmitic acid.

## 5 Conclusion

Our results indicate that including palmitic acid and a combination of palmitic, stearic, and oleic acids in proportions resembling those found in ruminal mixed bacteria improved ruminal fiber degradation. The improvement in fiber degradation is linked to changes in the rumen bacterial community, such as an increased relative abundance of *Prevotella* and *Fibrobacter*. Additionally, PA and PSO enhance the flow of short-chain fatty acids, such as acetate and propionate, which correlates with improved fiber degradation. We also observed that palmitic acid and a combination of palmitic, stearic, and oleic acids tended to increase microbial flow and modify membrane fatty acid composition. In contrast, stearic and oleic acids alone had minimal impact on fiber degradation and bacterial composition. Our data suggests that within the combinations of fatty acids tested in the supplemental fat, only when stearic and oleic acids are combined with palmitic acid, they can positively influence rumen fermentation and nutrient degradation.

## Data Availability

The original contributions presented in the study are publicly available. This data can be found here: 10.5281/zenodo.16753837.
