# Supplementary material for: Palmitic acid alone or combined with stearic and oleic enhances ruminal fiber degradation and alters microbiome composition
Source: Front Microbiol. 2025 Aug 15;16:1624738. doi: 10.3389/fmicb.2025.1624738 (PMC12394162; doi:10.3389/fmicb.2025.1624738)
Supplement: Supplementary file 1 [file Supplementary_file_1.docx]

Fernanda Batistel^1^*, Osvaldo Gonzalez^2^, Austin Sears^3^, Sharif Uddin Khan^1^, and Jonas de Souza^4^

^1^Department of Animal Sciences, University of Florida, 2250 Shealy Drive, Gainesville, Florida 32611, USA.

^2^Department of Animal Sciences, Utah State University, Logan, Utah 53706, USA.

^3^Department of Animal Sciences, Utah State University, Logan, Utah 53706, USA.

^4^Perdue Agribusiness, Salisbury, Maryland 21804, USA.

***Correspondence:**Fernanda Batistel

[fernandabatistel@ufl.edu](mailto:fernandabatistel@ufl.edu)


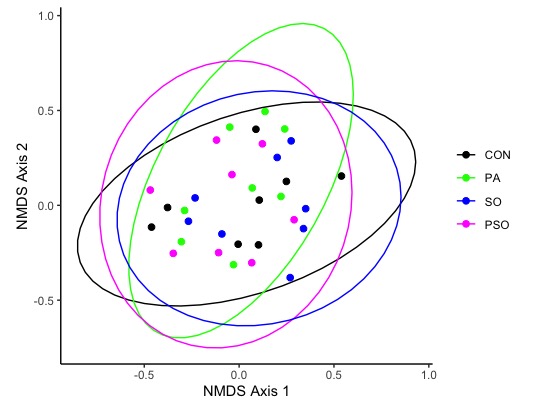


**Supplementary Figure S1.** Non-Metric multidimensional scaling (NMDS) plot of the Bray-Curtis similarity index of rumen microbial communities in response to supplemental combinations of palmitic, stearic, and oleic acid in continuous culture fermenters. The control (CON) was a basal diet composed of 50% orchardgrass hay and 50% concentrate (DM basis) without supplemental fatty acids. PA treatment supplied 1.5% of palmitic acid (% DM); SO treatment supplied 1.41% of stearic acid + 0.09% of oleic acid (% DM); PSO treatment supplied 0.48% of palmitic acid +0.95% of stearic acid + 0.075% of oleic acid (% DM).

**Supplementary Table S1.** Alpha diversity of microbial analysis to the supply of combinations of palmitic, stearic, and oleic acid in continuous culture fermenters.

| Item | Treatments^1^ | | | | SEM | *P*-value^2^ |
| --- | --- | --- | --- | --- | --- | --- |
|  | CON | PA | SO | PSO |  |  |
| n seqs | 46996 | 46985 | 46992 | 47020 | 38.4 | 0.15 |
| Sobs | 752 | 807 | 802 | 798 | 32.1 | 0.70 |
| Coverage | 0.998 | 0.998 | 0.998 | 0.997 | 0.005 | 0.84 |

^1^ The control (CON) was a basal diet composed of 50% orchardgrass hay and 50% concentrate (DM basis) without supplemental fatty acids. The control (CON) was a basal diet composed of 50% orchardgrass hay and 50% concentrate (DM basis) without supplemental fatty acids. PA treatment supplied 1.5% of palmitic acid (% DM); SO treatment supplied 1.41% of stearic acid + 0.09% of oleic acid (% DM); PSO treatment supplied 0.48% of palmitic acid +0.95% of stearic acid + 0.075% of oleic acid (% DM).

^2^*P*-values refer to the ANOVA results for the main effect of fatty acid treatment.

**Supplementary Table S2.** *P*-values associated with Permanova analysis of microbial similarity in response to the supply of combinations of palmitic, stearic, and oleic acid in continuous culture fermenters.

|  |  | Index | |
| --- | --- | --- | --- |
| Item | Test | Bray-Curtis | Jaccard |
| Treatment | PERMANOVA | 0.78 | 0.82 |

^1^The control (CON) was a basal diet composed of 50% orchardgrass hay and 50% concentrate (DM basis) without supplemental fatty acids. The control (CON) was a basal diet composed of 50% orchardgrass hay and 50% concentrate (DM basis) without supplemental fatty acids. PA treatment supplied 1.5% of palmitic acid (% DM); SO treatment supplied 1.41% of stearic acid + 0.09% of oleic acid (% DM); PSO treatment supplied 0.48% of palmitic acid +0.95% of stearic acid + 0.075% of oleic acid (% DM).
